# Induction of Hairy Roots on Somatic Embryos of Rhizoclones from *Typha domingensis* Seedlings

**DOI:** 10.3390/plants9121679

**Published:** 2020-12-01

**Authors:** Guadalupe Hernández-Piedra, Violeta Ruiz-Carrera, Alberto J. Sánchez, Alfonso Azpeitia-Morales, Graciano Calva-Calva

**Affiliations:** 1 Programa de Doctorado en Ecología y Manejo de Sistemas Tropicales, Universidad Juárez Autónoma de Tabasco, Carretera Villahermosa-Cárdenas Km. 0.5 S/N Entronque a Bosques de Saloya, Villahermosa 86150, Tabasco, Mexico; 2 Diagnóstico y Manejo de Humedales Tropicales, Universidad Juárez Autónoma de Tabasco, Carretera Villahermosa-Cárdenas Km. 0.5 S/N Entronque a Bosques de Saloya, Villahermosa 86150, Tabasco, Mexico; alberto.sanchez@ujat.mx; 3 Campo Experimental Huimanguillo, Instituto Nacional de Investigaciones Forestales, Agrícolas y Pecuarias, Huimanguillo, Km. 1 Carretera Huimanguillo-Cárdenas, Huimanguillo 86400, Tabasco, Mexico; azpeitia.alfonso@inifap.gob.mx; 4 Biotecnología y Bioingeniería, Centro de Investigación y de Estudios Avanzados del IPN, Avenida Instituto Politécnico Nacional 2508, Colonia San Pedro Zacatenco, Ciudad de México 07360, Mexico; gcalva@cinvestav.mx

**Keywords:** agrotransformation, cattail, rhizogenesis, somatic embryos, hairy roots

## Abstract

A protocol for the induction of hairy roots on somatic embryos of rhizoclones from *Typha domingensis* seedlings grown in hydroponic rhizotron systems was established for the first time. Rhizogenesis was induced through the agrotransformation of somatic embryos in oblong and scutellar states of development using the K599, LBA9402, and A4 strains of *Agrobacterium rhizogenes*. The transfection to the embryos was performed by cocultivation of rhizoclones on a Murashige and Skoog mineral medium at 50% strength (MS0.5), in the dark, at 28 ± 2 °C for 72 h. In contrast to nontransformed embryos that did not exhibit any root tissue, transformed embryos presented hairy roots that varied in number, length, and density depending on the bacterial strain, and K599 was the most effective strain. After analysis via optical microscopy, the transformed embryos were collected and transferred to fresh culture media supplemented with 400 mg mL^−1^ cefotaxime and 10 mg L^−1^ ascorbic acid. The efficiency of transformation and survival of the oblong and scutellar embryos were similar among the three bacterial strains. The results show that agrotransformation of somatic embryos of rhizoclones from *T. domingensis* is a novel and viable strategy for the generation of genetic transformants of *Typha* that have potential applications in bioremediation technologies.

## 1. Introduction

**Figure 1:**
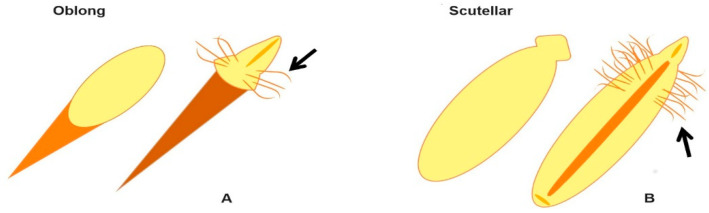
Schematic surface morphology of somatic embryos of *T. domingensis* in the oblong (**A**) and scutellar (**B**) stages, and nontransformed (left) and agrotransformed (right) stages. Both arrows shown the induction of hairy roots. For a complete histogenic model of the somatic embryos of *T. domingensis,* please refer to Hernández Piedra et al. [33].

The treatment of wastewater from water receptor ecosystems by using emergent aquatic macrophytes (EAM) plant species in phytoremediation-based processes is increasing as a result of the plasticity and physiological capacity of such plants to tolerate, accumulate, or remove both organic and inorganic environmental pollutants; moreover, this approach has led investigations of the genetic transformation of these species to improve the phytoremediation processes and the ecological recovery of the affected ecosystems [1,2,3,4,5,6] Typically, the plant species used for phytoremediation must be highly capable of adapting to the local environmental conditions of the polluted site, and as a consequence, stable genetic transformants from native species of the impacted sites are preferred [2,6]. Thus, to enhance the phytoremediation capability of *Typha domingensis* [7,8,9,10,11,12,13,14,15], and specifically its eutrophic water receptors [8,9,16], the aim of this work was to investigate the efficiency of the agrotransformation and survival of embryos in the oblong and scutellar stage (Figure 1) induced from in vitro rhizoclones grown in hydroponic rhizotron systems of an ecotype of *T. domingensis* Pers. (cattail). Although the genetic transformation of several EAM species has been achieved in both monocotyledonous and dicotyledonous species by using the bacterial genus *Agrobacterium* [3,6], which was later renamed as *Rhizobium* based on comparative 16S rDNA analyses [17], it is well known that, with exception of *T. latifolia* reported by Nandakumar et al. [18,19], the genetic transformation of the genus *Typha* remains a challenge because its capacity for transfection includes a narrow range of genotypes, and the utility of the technique is limited by the recalcitrance of many genotypes to regenerate transformed individuals [5,20,21]. In this work, the use of germinal lines or somatic embryogenesis systems to facilitate the transformations and to guarantee the regeneration of other monocotyledon species [22,23] were hypothesized to alleviate this problem. For example, the embryogenic callus of *Spartina alterniflora* were transformed with the *A. tumefaciens* LBA4404 strain to transport the organomercurial lyase, and mercuric reductase genes have led to the generation of stable genetic lines capable of resisting up to 500 μM HgCl_2_ [23]. Moreover, Mankin et al. [24] proposed the disarmed *A. rhizogenes* strain K599 as a candidate to induce stable transformed roots of *Arabidopsis thaliana*, *Zea mays*, *Lycopersicon esculentum,* and *Glycine max*, and the transformation of *Scirpus americanus* roots with the *A. rhizogenes* A4 strain produces clones capable of removing 3.8 and 1.2 times more Pb and Cr than those of nontransformed roots [25,26]. Nevertheless, regarding the genetic transformation of the *Typha* genus used in this work, we found only two reports in the literature [18,19], with both from Nandakumar et al., who reported the agrotransformation of *T. latifolia* callus tissue by cocultivation with *A. tumefaciens* EHA105 and LBA4404 strains. On the other hand, although we were unable to find reports about the genetic transformation of *T. domingensis*, it was chosen because it is a monocotyledonous EAM intercontinentally dispersed in the wetlands of Europe, Asia, and America continent [27,28]. In addition, it is a highly flood- and salt-tolerant species [9,16,29] that can grow in eutrophic water with anaerobic substrates [7,8] and can uptake heavy metals [9,15]. Furthermore, this species has been used in the floating treatment of wetlands for the phytoremediation of heavy metals from wastewater [10,11,12,13,14], oil-contaminated water [30], and water from wetlands containing high phosphate concentrations [7,31]. Additionally, in Mexico, *T. domingensis* is known as a cosmopolitan species that can be found in tropical wetlands [8,32] and has expanded its domain into eutrophic areas [16]. In a previous work, we reported that a callus tissue embryogenic line (YC1, yellow callus obtained with 0.5 mg L^−1^ 2,4-D) of *T. domingensis* was able to induce high proliferation of somatic embryos (SEs) in the oblong (SEo) and scutellar (SEsc) states of development [33]. In the present work, genetic transformation for the induction of hairy roots on SE of rhizoclones from *T. domingensis* seedlings by cocultivation with *A. rhizogenes* was investigated. The efficiency of three wild strains of *A. rhizogenes* was evaluated for the induction of hairy roots on SEs. The number, length, and density of the hairy roots were monitored on SEs infected with the *A. rhizogenes* K599, LBA9402, and A4 strains. The results offer, for the first time, a feasible protocol for transforming *T. domingensis* that will strengthen the physiological capacity of its root system with the goal of regenerating transgenic plants from transformed embryos opening the possibilities of further investigations of this plant in phytoremediation technologies for treatment of eutrophic water and soils.

## 2. Results

**Figure 2:**
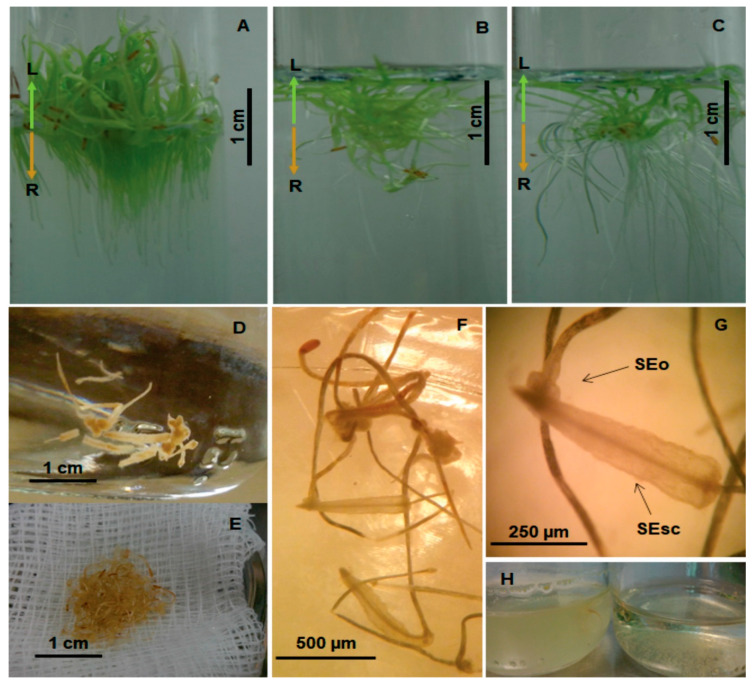
Induction of somatic embryos on rhizoclones from *T. domingensis* seedlings. Induction started with the in vitro germination of *T. domingensis* seeds in rhizotron systems with MS0.1 semisolid (**A**) or liquid (**B**) culture medium or with sterile purified water alone (**C**) for the establishment of rhizoclones for the induction of somatic embryos (**D**), harvested from single cultures (**E**). Those embryos showing scutellar (SEsc) and oblong (SEo) morphological stages (**F**,**G**) were transferred into a suspension of *A. rhizogenes* (**H**) for genetic transformation by agrotransfection. The arrows show the magnification of each somatic embryo in G imagen. L = Leaf, R = Roots.

Hairy roots on the surface of SEs of *T. domingensis* were successfully induced via agrotransformation of in vitro rhizoclones grown in hydroponic rhizotron systems (Figure 2). Based on the natural environmental and nutritional conditions of the sites where wild *T. domingensis* grows [34], three treatments for the selection of a culture medium that can to support the rhizotron system (Figure 2) were implemented to establish adequate rhizoclone development via the in vitro germination of seeds. These treatments included growth on MS0.1 semisolid culture medium (Figure 2A), growth in liquid MS0.1 medium without growth regulators (Figure 2B), or growth in purified sterile water alone (Figure 2C); in addition, all the treatments included ascorbic acid. Both liquid systems produced friable tissue (Figure 2B,C) with crumbly rhizoclones suitable for the induction of embryogenic callus with embryos in several developmental stages (Figure 2D), as previously reported [33]. Moreover, further experiments revealed that the rhizoclones induced in rhizotron systems, incubated in the dark, yielded a large amount of unpigmented embryos in the scutellar and oblong morphological stages of development (Figure 2D–F), which were suitable for studying the induction of hairy roots by cocultivation with *A. rhizogenes* (Figure 2H).

**Figure 3:**
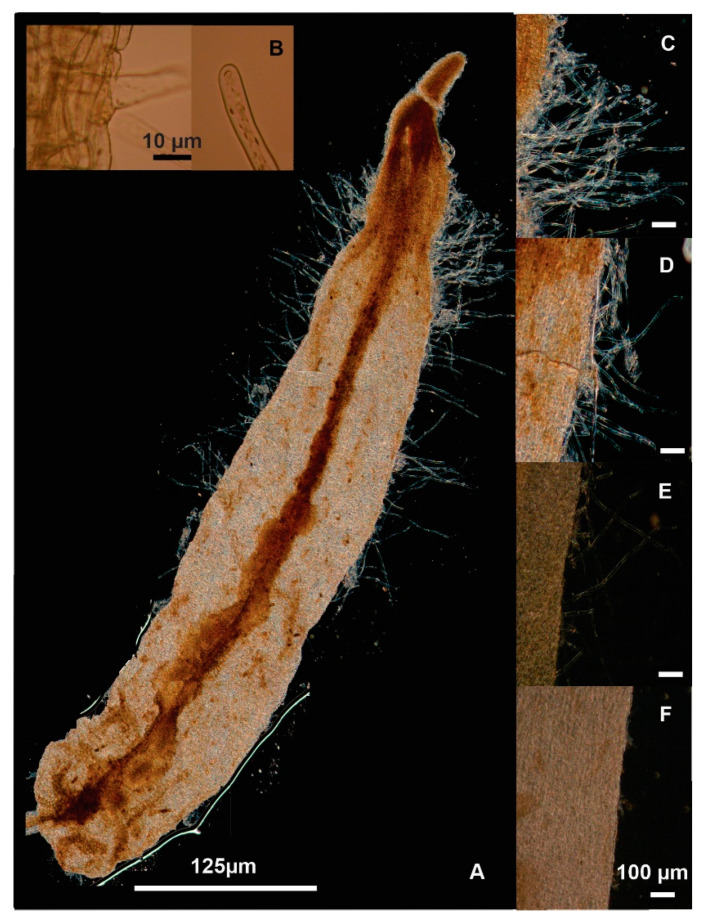
Agrotransformed somatic embryos of *T. domingensis* after 28 days of coculture with *A. rhizogenes* K599 (**A**), epidermal tissue with hairy root and a hairy root tip emerging from the infected embryo (**B**), hairy roots density on the surface of somatic embryos infected with *A. rhizogenes* K599 (**C**), and A4 (**D**) and LBA9402 (**E**) compared with a non-infected control embryo (**F**).

Three wild *A. rhizogenes* strains were tested for hairy roots induction on the SEs of rhizoclones from *T. domingensis* seedlings through genetic transformation of a SE line (YC1) in the oblong and scutellar states of development; these strains included the K599, LBA9402, and A4 strains (Figure 3). The transformed embryos clearly showed the presence of hairy roots (Figure 3A–E) emerging from single cells (Figure 3B), whereas the nontransformed embryos did not show root tissue (Figure 3F). Hence, transfection to the embryos by coculture in MS0.5 medium in the dark with any of the three bacteria was successful, although the number, density, and length of the hairy roots were significantly different between the bacterial strains (Table 1). The transformed embryos showing the presence of hairy roots were collected and transferred to a fresh culture medium with 400 mg mL^−1^ cefotaxime and 10 mg L^−1^ ascorbic acid, a compound that is indispensable for the survival and health of embryos. 

**Figure 4:**
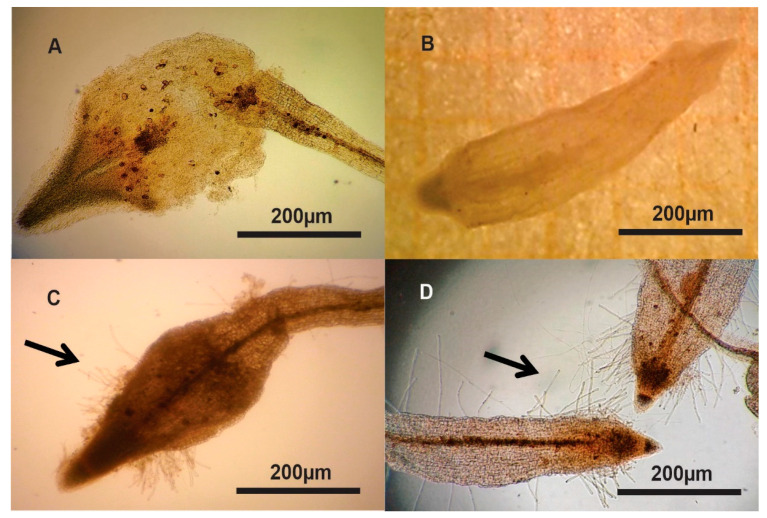
Surface of nontransformed (**A**,**B**) and agrotransformed somatic embryos in the oblong (**A**,**C**) and scutellar (**B**,**D**) stages established from the embryogenic rhizoclone after 28 days of transfection with the K599 *A. rhizogenes* strain. Note the higher abundance of hairy roots (arrows) on the apical tips of the agrotransformed tissue (**C**,**D**) compared with the absence of adventitious roots on the nontransformed tissue (**A**,**B**).

The infectivity of *A. rhizogenes* was evaluated by examining the typical appearance and morphology of the hairy roots that emerged on the treated embryos compared with the nontransformed tissue (Figure 4) as suggested in numerous reports [2,4,5]. As observed, the formation of the hairy roots on the embryos was due to transfection by using any of the three *Agrobacterium* strains, but hairy roots were not visible on the noninfected embryos. Additionally, the structure and morphology of the transformed hairy roots were similar and independent of the bacterial strain used (Figure 3 and Figure 4). In general, the hairy structures originated from single epidermal cells (Figure 3B) that looked like tubular unicellular extensions in which the cells contained peripheral cytoplasm and a large central vacuole as reported for classical transformed hairy root tissue [5]. No similar root tissue was observed on the treatment with control embryos (Figure 4A,B). It should be noted that greater abundance of hairy roots was observed in the apical and middle areas of the embryos (Figure 4C,D), which are areas reported to have higher abundance of meristematic cells [4,5].

The effects of the *A. rhizogenes* strain, and the morphological stage of the SE, on the agrotransformation process were evaluated and the results revealed that both factors affected the transformation frequency, survival, and hairy density (Table 1). Evidently, the efficiency of transformation for both the oblong and the scutellar embryos was similar for each of the three bacterial strains; however, survival was affected by the Agrobacterium strain but not the morphological stage. Interestingly, the SEs infected with the K599 strain produced a greater number, length, and density of hairy structures than those infected with the LBA9402 and A4 strains, and these results are consistent those discussed above for Figure 3C. However, in general, the *Agrobacterium* strains did not affect the effectiveness of the agrotransformation process of either the SEo or SEsc embryos (Kruskall–Wallis test; *p* > 0.05). It should be noted that the SEo stage showed lower frequencies of transformation than the SEsc stage, which affected the total number of SEs transformed (SEo plus SEsc). This finding is consistent with the observation that the efficiency of transformation was highly variable between the morphological stage of embryos (25–88%). Notably, frequent contamination was observed during cocultivation with the A4 and LB9402 strains (Figure 2H, left flask), which may have resulted in the significantly different and lower level of survival of the SEo stage of each bacterial strain (92–54%) as confirmed by the Kruskall–Wallis test (*p* = 0.09), and the significantly different results for the SEsc stage (Kruskall–Wallis test; *p* = 0.003). Notably, the K599 strain stood out in all embryos regarding the frequency and survival of the transformed embryos, as well as the number of hairy roots (Table 1). Finally, the frequency of transformation in total SEs was substantially greater for the K599 strain. Moreover, the survival of the SEsc and the total SEs was significantly greater (Kruskall–Wallis test; *p* = 0.002) with the K599 strain.

## 3. Discussion

The SEs of *T. domingensis* developed typical hairy roots through the transfection of rhizoclones with the *A. rhizogenes* K599, LBA9402, and A4 strains. The phenotype of these hairy roots was similar to the phenotype of hairy roots reported in the transgenic embryos of *Bouteloua gracilis* [35]. The virulence was different for each of the three *A. rhizogenes* strains; however, the K599 strain stood out in terms of its hypervirulence, and the A4 strain stood out in terms of its virulence. Hypervirulent strains of *Agrobacterium* spp., have been proven effective in other species, such as *Cyclamen persicum,* in which embryogenic callus cocultivated with the *A. tumefaciens* EHA105 hypervirulent strain resulted in a transformation efficiency twice as high as that achieved with the *A. tumefaciens* LBA4404 virulent strain [36]; moreover, similar results were achieved with the *A. rhizogenes* K599 and LBA9402 strains in this study. The K599 strain, which is equivalent to the NCPPB2659 cucumopine biovar, harbors the pRi2659 plasmid (ADN-T) that transports the aux1 and aux2 genes for auxin autotrophic cell division and biosynthesis and the onset of hairy roots, including root loci oncogenic genes (mainly *rol A, B,* and *C* genes), and other genes with unidentified function, such as open reading frames [24,37,38]. The genes cluster *aux* and *rol A, B,* and *C* play a part in the infection and induction of hairy roots [39].

The root hairs in the SEs of *T. domingensis* in both the oblong and scutellar embryogenic states developed on the epidermis towards the apex and middle of the cotyledon area. Similarly, the root hairs on the root of *A. thaliana* emerge from epidermal cells and form short hairs on the basal area oriented towards the root apex [40], thus showing acropetal development of root hairs, which is characteristic of axonomorphic roots [41].

The critical requirements to confirm the genetic agrotransformation of a plant tissue include the identification of transformed cells or target tissue, evidence of the presence and/or expression of the transferred T-DNA in the nuclear genome, and the regeneration of transgenic plants [42,43]. In this work, the first requirement was confirmed by the selection of embryos showing hairy roots with the typical morphological pattern of such tissue, such as branching and growth on the medium without plant hormones. The biological effect and confirmation for the genetic transformation by the presence of the transferred DNA (T-DNA) of *A. rhizogenes* to the target plant tissue is still under study, as there are certain limitations in the case of recalcitrant species [39,44]. The infectivity of the strains has been confirmed via morphologic markers of the hairy phenotype in the *Agrobacterium*-treated SEs [35] as well as by the coculture technique performed in this study. Similar to the study that induced transgenic embryos of *Bouteloua gracilis*, polymerase chain reaction (PCR), Southern blot, and Northern blot analyses are commonly used to confirm the presence of the *rol A* genes in hairy SEs; however, these molecular techniques are not infallible for confirming genetic transformation. For example, the secondary embryos of *Coffea canephora* transfected with the A4 rhizogenic wild strain molecularly revealed a stable transformation, but without the hairy phenotype [45]. In contrast, the presence of the hairy root morphological phenotype in agrotransformed tissue with *A. rhizogenes* wild strains provides proof for the success of the genetic agrotransformation [20,24,35,37,44,45,46]. 

Knowledge of the interaction between *Agrobacterium* and the structure and composition of the outer cell wall of the protodermic cells that cover SEs is limited [47]. The transfective susceptibility of *A. tumefaciens* in root hairs may be explained by the biochemical changes that take place on the cell surface [48]. The biochemical mechanism was revealed by the presence of polar binding polysaccharides and an unknown receptor molecule based on the greater number of adhered bacteria. As previously reported [48], the adherence of *Agrobacterium* to the surface of embryos might have been improved by the acid pH (5.5) of the culture medium used in the coculture, the absence of accessory structures such as cutin in this type of tissue [47], or the addition of ascorbic acid to the culture system [49]. In addition, *Agrobacterium* has been reported to show enhanced virulence in the presence of phenolic compounds [50], such as those produced by *T. domingensis* embryos [33]. Thus, consistent with these results, the acid pH, addition of ascorbic acid, and liquid state of the coculture medium all favored biochemical adherence of the bacteria to the embryo surface, thereby enhancing the agrotransformation process.

The high variability in the frequency of transformation and survival of the transformed tissue observed in this work agree with studies of transformations mediated by *Agrobacterium* of the monocotyledon species, such as *Triticum aestivum* (wheat), *Oryza sativa* (rice), and *Zea mays* (maize), for which high variable frequencies of transformation have been reported [51,52]. Additionally, a general variability for the efficiency of the agrotransformation of immature (14.9%) or mature (9.8%) embryos of wheat, and low efficiencies of transformation of zygotic embryos of maize (1.21–10.96%) with the *A. rhizogenes* K599 strain, with or without extra copies of virulence genes, have been reported [25]. Furthermore, high variability in the transformation efficiency has also been reported by using the electroporation technique for some rice species, and it produced more than 80% efficiency for some varieties but less than 2% for others [53].

Overall, since hairy roots can emerge only from single transformed cells expressing of transferred T-DNA [42,43,54], the present results suggest that the hairy root specific phenotype may become a practical morphologic feature for the early identification of an agrotransformation event in embryos because it might not require additional molecular or biochemical techniques. In addition, this approach provides a simple and rapid method for the genetic transformation of *Typha* species through the coculture of embryos with *Agrobacterium*, although molecular techniques should be used to validate the relationship between the hairy phenotype and the genetic transformation success. Such strategy can be combined with the current machine learning algorithms technology, which have emerged as promising tools for prediction of hairy root cultures, among other in vitro cultures types [55,56], along with the optimization of environmental conditions to achieve maximum productivity and efficiency of hairy root cultures of *Typha domingensis* as a valuable plant for biotechnological applications. On the other hand, it is still uncertain why the presence of ascorbic acid enhanced the survivance and health of embryos as previously reported [36]; however, this compound has been used in genetic transformation events of several plant species due to its antioxidant properties [57,58] Additionally, the frequency of infection with the *A. rhizogenes* strains used in this work should be studied for the transformation of other dicotyledonous species. Moreover, although the regeneration of transformed plants is still a pending issue in this work, whether the agrotransformation of other plant species by using the present approach results was successful, this could be a good advance in the area of plant biotechnology. 

In conclusion, infection of *T. domingensis* somatic embryos, in the oblong and scutellar stage of development, with the three *A. rhizogenes* strains was an efficient strategy for inducing hairy roots, and the K599 strain was the most effective. The results provide new insights in the area of plant biotechnology by providing a new protocol for genetic transformation of plant species recalcitrant to genetic transformations. Additional studies are necessary to molecularly confirm the transgenic nature of the tissues and the regeneration of transformed plants potentially useful for biotechnological applications for the removal of pollutants from hypereutrophic wastewater and the production of natural compounds. 

## 4. Materials and Methods 

### 4.1. Germination of Typha Seeds and Establishment of Rhizoclones on Somatic Embryos (SEs)

Seeds were isolated from the achenes of *T. domingensis* and germinated in rhizotron systems to establish rhizoclones. The achenes were collected from the tropical wetlands of Villahermosa, Tabasco (17°59′9.91″ N and 17°57′48.98″ E), Mexico, and deposited in the dry plant collection of the Herbarium in the Universidad Juárez Autónoma de Tabasco (UJAT-Herbarium 35227). The seeds used for this work were stored in a desiccator at room temperature, and 5–15 seeds were sowed in each rhizotron unit (sRU) established in test tubes with 20 mL of Murashige and Skoog basal medium [59] at one-tenth ionic strength (MS0.1), in either semisolid (agar 4% *w*/*v*) or liquid format with pH 6.5 and formulated with MS vitamins, without growth regulators in an autotrophic regime. Additionally, a sRU with deionized sterile water was prepared as a control. The rhizotron systems in the semisolid medium were covered with a water film of 1 cm. After 15 days germination, the root system of seedlings with 1 cm length and with similar development (pair of leaves, radicle with adventitious root) were separated from the caulinar base and transferred into a new sRU for the establishment of rhizoclones (Figure 2). Individual roots were positioned vertically on the surface of the semisolid culture medium, avoiding injuries, and were incubated under photoperiod (16/8 h light/darkness) by using cold white light (Philips, USA), at a photon flux density of 20 μmol photons m^−2^ s^−1^ (Quantum Light Meter; Spectrum Technologies, Aurora, IL, USA), and a temperature of 28 ± 2 °C. The rhizoclones were used for the induction of SEs by incubation in the dark in MS culture medium at half ionic strength (MS0.5), and supplemented with vitamins, sucrose (3% *w*/*v*), ascorbic acid (10 mg L^−1^), and 0.5 mg L^−1^ 2,4-D, as described previously [33]. The SEs in the oblong (SEo) and scutellar (SEsc) stages of development were isolated from the embryogenic line YC1 of *T. domingensis* that was established and maintained as described above.

### 4.2. Agrobacterium Rhizogenes Strains

The *A. rhizogenes* K599 (cucumopine-type), LBA9402 (agropine-type), and A4 (agropine-type) strains were provided by the Metabolic Engineering group at CINVESTAV-IPN. The bacterial strains were kept at 4 °C in Luria-Bertoni semisolid culture medium (LB) at pH 7. The bacteria were first activated by transferal to the LB liquid culture medium using commercial yeast extract and incubation in an orbital shaker at 30 °C and 130 rpm for 48 h. The concentration of bacteria for infection of the embryogenic tissue was adjusted at DO_600_ 0.8–1 to proceed to inoculation as described by Jimenez-Antaño et al. [60].

### 4.3. Induction of Transformed Hairy Roots on SE of Typha Domingensis

The inoculation of the SE of *T. domingensis* with the *A. rhizogenes* strains was performed by coculture. A bacterial suspension with 48 h of growth was mixed at a ratio of 1:5 (final volume 30 mL) with the *T. domingensis* embryo MS0.5 medium culture as described for embryogenic induction, although the medium was not supplemented with phytoregulators. The mixture was incubated in the dark, at 28 ± 2°C, for 72 h, and it was stirred constantly (130 rpm, 60 min) (*n* = 4). The embryogenic callus and the SEo and SEsc in the embryogenic mixture of each culture unit were filtered through a sterile gauze, transferred to 30 mL of MS0.5 medium with 400 mg/L cefotaxime (*n* = 4) to eliminate the residual *A. rhizogenes*, and kept under the previous environmental conditions for 28 days.

### 4.4. Experimental Model

The effect of the *A. rhizogenes* K599, LBA9402, and A4 strains on the efficiency of transformation of *T. domingensis* EL-YC1 was studied. The observational unit was the viable SEs (*n* = 40 per treatment), which were randomly sampled from the MS medium with cefotaxime. The transformed embryos were separated following a previously reported protocol [33]. The survival, hairy morphology, and transformation frequency of all viable SEs, SEos, and SEscs were analyzed at the end of the experiment.

### 4.5. Agrotransformation Quantification

Survival was estimated considering the excessive growth of bacteria [51] since the number of viable embryos per treatment decreased with the three strains. The visual indicators used to calculate the frequency of agrotransformation were the hairy SEo and SEsc phenotypes observed under a stereomicroscope (Carl Zeizz^®^, Göttingen, Germany). The magnitude of hairiness in the SEo and SEsc stages was quantified through an image analysis (*n* = 3) of the number (No.), length (μm), and density (No. μm) of the hairy units [60]. The SE images were digitized using an Axio Scope A1 optical microscope (Carl Zeizz^®^) equipped with an AxioCam ERc5s camera (Carl Zeizz^®^). The fields of view of interest were selected using the 10×, 40×, and 100× + 1.25× objective lens in the optovar (Carl Zeizz^®^) and the SEN/2011 program (Carl Zeizz^®^ Microscopy GmbH, 2011).

### 4.6. Statistical Analyses

Kolmogorov-Smirnov and Cochran C tests were used to verify the assumptions of normality and homoscedasticity of the data. The Fisher’s least significant difference test (LSD) or the Kruskal–Wallis H test were used to estimate the statistical difference between treatments by using a significance level of 0.05. Statistica V8 (Stat Soft Inc., Tulsa, OK, USA) software was used for statistical analyses.

## Figures and Tables

**Table 1:** Effects of the *Agrobacterium rhizogenes* strain on the transformation, survival, and hairy density of *Typha domingensis* somatic embryos.

		Strain of *Agrobacterium rhizogenes*			Kruskal–Wallis	
Somatic Embryo	Dependent Variable	K599	LBA9402	A4	H	P
Oblong (SEo)	Frequency of transformation (%)	25 ± 12	20 ± 13	14 ± 16	0.3	0.86
	Survival (%)	92 ± 12	71 ± 12	54 ± 12	4.7	0.09
Scutellar (SEsc)	Frequency of transformation (%)	88 ± 8	82 ± 9	79 ± 10	0.6	0.73
	Survival (%)	93 ± 8 ^a^	65 ± 9 ^b^	52 ± 8 ^b^	10.9	0.003
Total SE	Frequency of transformation (%)	68 ± 8	59 ± 9	57 ± 11	0.8	0.68
(SEo + SEsc)	Survival (%)	93 ± 7 ^a^	68 ± 7 ^b^	53 ± 7 ^b^	15.7	0.002
	Hairy Number (No)	39.0 ± 1.2 ^a^	9.0 ± 7.5 ^bc^	22.3 ± 9.7 ^ab^		0.01
	Length (µm)	363.3 ± 39.6	165.2 ± 82.7	306.0 ± 34.2	3.6	0.30
	Density (No/mm)	21.51 ± 3.3 ^a^	5.64 ± 4.7 ^b^	13.04 ± 5.6 ^ab^		0.02

The data represent the means ± standard error of the treated embryos. Different letters in columns denote a group of means that are significantly different (*p* < 0.05). H = test statistic for the Kruskal–Wallis H test; P, *p*-value for the Kruskal–Wallis test or from the F = Fisher LSD test.
